# Role of central endpoint adjudication and challenges in trials on neonatal sepsis—a case of ProSPoNS trial

**DOI:** 10.1186/s13063-024-08298-0

**Published:** 2024-07-05

**Authors:** Anju Pradhan Sinha, Dilip Raja, Kamlesh S. Mahajan, Piyu Sharma, Subodh S. Gupta, Ramesh Poluru, Anand S. Kawade, Girish Dayma, Sunil Sazawal, Ashish Bavdekar, Sailajanandan Parida, Sushma Nangia, Abhishek V. Raut, Adhisivam Bethou, Prabhabati Devi, Makrand Gorpade, Tharika Fatima, Rashmita Nayak, Arti Kapil, Mohd. Azam, Pearlin A. Khan, Ravindra Mohan Pandey, Narendra Kumar Arora, ProSPoNS Study Group, Dixit Prajapati, Apoorva Mathur, Pankaj Gupta, Payal Kumari

**Affiliations:** 1 Division of Reproductive, Child Health and Nutrition (RCN), Indian Council of Medical Research (ICMR) Headquarters, V Ramalingaswami Bhawan, Ansari Nagar, New Delhi, Delhi, 110029 India; 2 Department of Community Medicine, Dr. Sushila Nayar School of Public Health, Mahatma Gandhi Institute of Medical Sciences (MGIMS), Sewagram, Wardha, Maharashtra 442102 India; 3 The International Clinical Epidemiology Network (INCLEN) Trust International, F-1/5, 2Nd Floor, Okhla Industrial Area Phase - 1, New Delhi, Delhi, 110019 India; 4 Department of Pediatrics, KEM Hospital Research Centre, 489 Rasta Peth, Sardar Moodliar Road, Pune, Maharashtra 411011 India; 5 Department of Neonatology, Jawaharlal Institute of Postgraduate Medical Education and Research (JIPMER), Dhanvantri Nagar, Gorimedu, Puducherry, 605006 India; 6 Centre for Public Health Kinetics (CPHK), 214 A, Vinoba Puri, Lajpat Nagar-II, New Delhi, Delhi, 110024 India; 7 Neonatal Health & Human Nutrition, Asian Institute of Public Health (AIPH), 8A, Unit-6, Ganga Nagar (Near Raj Bhawan), Bhubaneswar, Odisha 751001 India; 8 Department of Neonatology, Lady Hardinge Medical College and Associated Kalawati Saran Children’s Hospital (KSCH), Near Gole Market, Central Dist. New Delhi, Delhi, 110001 India; 9 Department of Microbiology, All India Institute of Medical Sciences (AIIMS), New Delhi, Delhi, 110029 India; 10 Department of Biostatistics, All India Institute of Medical Sciences (AIIMS), New Delhi, Delhi, 110029 India

**Keywords:** ProSPoNS, Neonatal, Sepsis, Clinical trial, Adjudication

## Abstract

Despite progress in reducing the infant mortality in India, the neonatal mortality decline has been slower, necessitating concerted efforts to achieve Sustainable Development Goal-3. A promising strategy aiming to prevent neonatal sepsis in high-risk, vulnerable, low birth weight neonates through an innovative intervention includes probiotic supplementation. This article communicates the decision by the ProSPoNS trial investigators to establish a Central Endpoint Adjudication Committee (CEAC) as an addendum to the protocol published in *Trials* in 2021 for the purpose of clarifying the primary outcome. In the published protocol, the study hypothesis and primary objective are based on “sepsis,” the primary outcome has been specified as sepsis/PSBI, whereas the sample size estimation was performed based on the “physician diagnosed sepsis.” To align all the three above, the investigators meeting, held on 17th–18th August 2023, at MGIMS Sevagram, Wardha, deliberated and unanimously agreed that “physician diagnosed sepsis” is the primary study outcome which includes sepsis/PSBI. The CEAC, chaired by an external subject expert and members including trial statistician, a microbiologist, and all site principal investigators will employ four criteria to determine “physician diagnosed sepsis”: (1) blood culture status, (2) sepsis screen status, (3) PSBI/non-PSBI signs and symptoms, and (4) the clinical course for each sickness event. Importantly, this clarification maintains consistency with the approved study protocol (Protocol No. 5/7/915/2012 version 3.1 dated 14 Feb 2020), emphasizing the commitment to methodological transparency and adherence to predefined standards. The decision to utilize the guidance of a CEAC is recommended as the gold standard in multicentric complex clinical trials to achieve consistency and accuracy in assessment of outcomes.

**Trial registration**

Clinical Trial Registry of India (CTRI) CTRI/2019/05/019197. Registered on 16 May 2019.

## Background

A protocol has been published for the ongoing phase III multicenter randomized double-blind placebo-controlled trial (ProSPoNS trial) evaluating the role of probiotics (Vivomixx) in the prevention of neonatal sepsis in 0–2-month-old Indian infants [1]. The trial is based on our previous pilot study that enrolled 1340 low birth weight neonates [2], showing an overall 21% non-significant reduction in the incidence of suspected sepsis diagnosed by field investigators using the possible serious bacterial infection (PSBI) (definition of WHO/UNICEF) [3] to detect suspected sepsis in the trial. However, in a non-pre-specified sub-group analysis among infants 1.5–2.00 kg, a 71% reduction in the incidence of sepsis in the intervention arm was observed. These results formed the rationale for the current trial, aiming to look for conclusive evidence of the potential benefit of the probiotic intervention. The technical advisory group (TAG) of the Indian Council of Medical Research (ICMR) suggested planning and implementing a larger trial with a sufficient sample size and a specific definition of the primary outcome “sepsis” since the PSBI definition used earlier was considered lacking in specificity. Subsequently, an application to the UKRI JGHT call 8 was made with success [4].

In the ProSPoNS trial, we opted for a strict definition of the primary outcome “sepsis” defined as one or more clinical signs suggestive of sepsis with a microbial isolate on blood culture or a neonate with sterile blood culture with at least 2 sepsis screen markers being abnormal (CRP > 12 mg/L, absolute neutrophil count < 1500/mm^3^, TLC < 5000/mm^3^, ESR > 15 mm, immature to total neutrophil ratio > 0.2).

Our second primary outcome in the trial was a possible serious bacterial infection (PSBI) as defined by WHO UNICEF. We used the data on “physician-diagnosed sepsis” [2] from the pilot trial to calculate the sample size for the ProSPoNS trial.

Secondary outcomes include (1) stool colonization patterns at baseline day 0, day 21, and 60 (end of the study) in a subsample. (2) Death and late-onset sepsis: comparison of all-cause deaths and late-onset sepsis between the intervention and control groups. (3) Clinical severe infection: one or more clinical signs—not feeding well, fever (temperature ≥ 38 °C), low body temperature (< 35.5 °C), severe chest in-drawing, movement only when stimulated as confirmed by the study physician. (4) Critical illness: one or more of clinical signs—convulsions, unable to feed at all, no movement on stimulation, unable to cry, bulging fontanelle, and cyanosis as confirmed by the study physician. Lastly, the cost-effectiveness/utility of the probiotics intervention vs control is being taken up as a sub-study to help inform policy for the prevention of neonatal sepsis.

The protocol [1] outlined the following:

- The IP administration compliance check and safety follow-up/screening for morbidities will be conducted in the community by field workers trained on IMNCI (2019) [5] guidelines.
- The participant would be followed up daily in the first week of life, thrice per week during weeks 2–4 of life, and weekly once in the second month of life.
- Field workers will be trained to screen and detect sick infants as per PSBI protocol. In case of any complaints, the field worker will accompany the parents with their infant(s) to the site hospital for further examination by the study investigator.
- A sepsis screen and blood culture would be performed to diagnose neonatal sepsis and appropriate treatment would be provided as per the hospital or the study protocol.

In this article, we communicate the decision of the ProSPoNS trial investigators to form a Central Endpoint Adjudication Committee (CEAC) as an addendum to the original published protocol in “*Trials* 2021.” We explain the reasons for coming to this decision by highlighting the challenges faced in trial implementation, the heterogeneity across sites in diagnosis of sepsis, and the academic variability in the definition of sepsis in the neonatal/young infant population.

## Definition of neonatal *sepsis*

Neonatal sepsis is commonly termed as an infection involving the bloodstream in newborn infants less than 28 days old. Diagnosis of sepsis in newborns is not easy due to the lack of a uniform definition of sepsis, unlike in children and adults where they are labeled as either “culture positive sepsis” or “clinical sepsis” [6]. The newborns suspected of sepsis based on maternal or peri-natal risk factors may be administered antibiotics, but their clinical course, sepsis screen and blood cultures if not suggestive of sepsis, are then labeled as “no sepsis” after 3 days.

Researchers/academicians recognize the problem of defining neonatal sepsis and lay down ground rules/SOPs [7, 8] for a working definition in studies and have discussed cases labeled as “culture negative sepsis” (suspected cases where despite sample collection bacteria may not grow because of several factors such as the timing of collection, bacterial load, and prior antibiotic consumption). The term “culture negative sepsis” has been explained based on the following criteria given below:

1. Symptomatology suggestive of sepsis or at least two maternal risk factors.
2. Individual sepsis screen markers namely total leucocyte count (TLC) and absolute neutrophil count (neutropenia) were considered. The ability of C-reactive protein (CRP) as a marker of late-onset sepsis (LOS) remains controversial in literature [9].
3. The clinical course of the disease where there is no other explanation for the symptoms can then be attributed to sepsis.

At the beginning of the study, it was assumed that blood culture-positive sepsis is the gold standard method of diagnosing neonatal sepsis. However, on completion of the microbiological tests, results yielded 30–40% of cases of culture positives. Therefore, it is not incorrect to say that it cannot reliably rule out sepsis. Reported rates of “culture-negative” or “suspected” sepsis vary widely in the literature. While some experts advocate considering sepsis evaluations completed after 48–72 h of negative blood cultures, data available from two large randomized controlled trials (RCTs) in recent years [10, 11] show culture-negative sepsis rates of 56% and 46%, respectively. By reviewing all morbidity cases individually, the CEA committee would take into consideration the culture negative reports and the contaminants with a balanced view.

## Rationale for central endpoint adjudication

The protocol specifies that if any illness is detected by a field investigator during follow-up, he should assess and classify the event and immediately escalate the event to the study physician. The study physician will manage the event as required, confirm the outcome classification, and suggest if blood sample collection is required or not. However, field implementation brought to light the reality that this algorithm of event referral and classification was not followed uniformly due to various reasons such as the parents being unwilling to visit study hospital after referral of their child, the parents were reluctant to visit the study site for minor complaints such as only fever, or in some cases the parents refused a blood sample collection of their child for such complaints. In other scenarios, the parents preferred taking treatment for their child from private practitioners.

In some scenarios where the participant went to private practitioners, sepsis testing was done irrespective of the protocol requirement and these samples tested positive without any clinical signs or symptoms. The proportion of COANS sepsis was observed to be high. There were instances where the blood culture tested positive when there was no clinical correlation with sepsis. Due to the above reasons, the rules set within the protocol may not hold true in all cases. Therefore, to eliminate under-reporting or over-reporting of events and accurately documenting the outcome as “sepsis,” the process of clinical end point adjudication is being adopted as a mid-course correction in the study.

## Central Endpoint Adjudication Committee for final primary outcomes

Central adjudication plays a key role in achieving consistent, accurate, independent, unbiased, and blinded evaluation of suspected clinical events reported by investigators in multicenter/large clinical trials [12]. It helps in the prevention of misclassification of outcomes in clinical trials that can lead to biased estimates of treatment effect and reduced power. Ensuring appropriate adjudication methods to minimize outcome misclassification is therefore, essential [13]. The adjudication committee will look at these cases in an unbiased and blinded manner.

In the course of trial implementation, it was felt that clarity about the primary study outcome definition needed to be documented explicitly before study completion or unmasking of the trial data. In the published protocol [1], the study hypothesis and primary objective are based on “sepsis,” the primary outcome has been specified as sepsis/PSBI, whereas the sample size estimation was performed based on the “physician diagnosed sepsis.” To align all the three above, the investigators meeting, held on 17th–18th August 2023, at MGIMS Sevagram, Wardha, deliberated and unanimously agreed that “physician diagnosed sepsis” is the primary study outcome which includes sepsis/PSBI. We propose a central endpoint adjudication process in order to align the primary objective and outcome with the stated hypothesis, and for enumeration of the primary outcomes listed across all six sites of the trial.

“Physician diagnosed sepsis” will be decided by the Central Endpoint Adjudication Committee (CEAC) based on four criteria for every event of sickness in a study participant namely blood culture status, sepsis screen status, PSBI/non-PSBI signs and symptoms, and the clinical course during the event of sickness including use of antibiotics.

The CEA committee will be chaired by one external subject expert and shall consist of all site PIs, trial statistician, and microbiologist. The CEA committee will go through every event of sickness based on these four criteria and make a decision regarding whether that event of sickness would be classified as “physician diagnosed sepsis” or not.

The above clarification does not make any change to the approved study protocol (Protocol No. 5/7/915/2012 version 3.1 dated 14 Feb 2020).

## The process of adjudication

The preparatory activity for adjudication involves data cleaning and finalizing the data to be shared with the adjudication committee. It will be followed by creating a data set with the details of participants and all the episodes of morbidities as subfolders in chronological order. The aim of creating a data set is to summarize and provide a complete clinical course of the event as well as the participant. All the forms (adverse event (AE), serious adverse event (SAE), follow-up by field investigator (FUP), study physician assessment form (SPF), concomitant medication form (CONMED), investigational product compliance (IPC), maternal history, and birth history) related to a particular event, especially the outcome-related variables of the data, would be retrieved and linked to generate a final summary/narrative of the cases. A statistical programming language “R” would be used to complete this activity.

The adjudication process involves three steps. In the first step, a team of the site investigators will assess their own data; they will revisit, assess, and finalize the classification of the outcomes. In second step, the site data of one site will be independently assessed by a team of another site investigators and the outcomes will be classified. Following the first two steps, there will be certain cases where the classification of outcomes by both the investigators will be in agreement and a few cases where it will be in disagreement. The disagreement will be resolved in third step, where the CEA chairperson will review all the discordant cases and finalize the outcome classification.

**Fig. 1:**
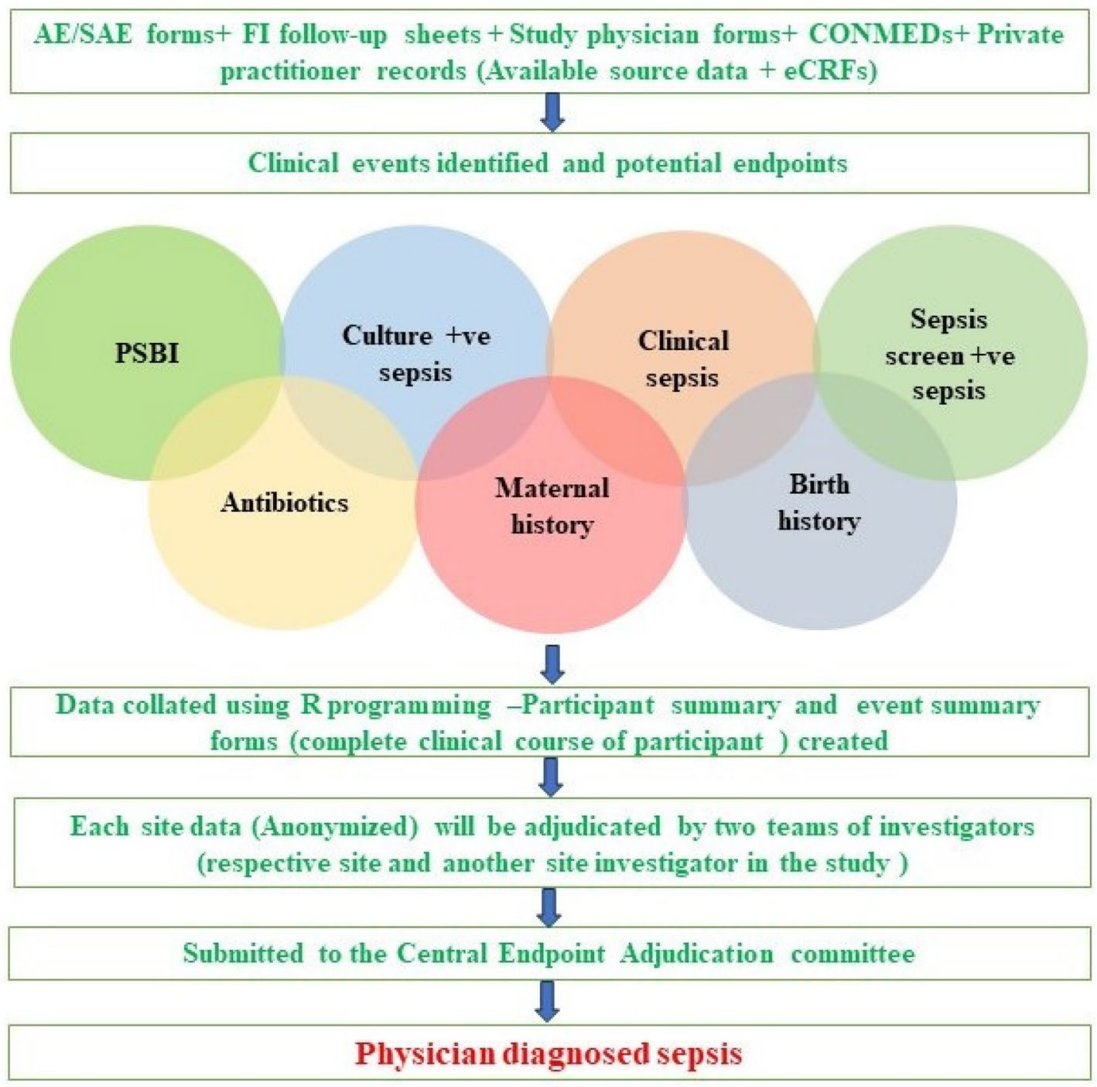
Adjudication process of ProSPoNS trial (CONMEDs, concomitant medications; SP, study physician form (designation wrt ProSPoNS study); SAE, serious adverse event; eCRFs, electronic case record forms; PSBI, possible serious bacterial infection; CEAC, Central Endpoint Adjudication Committee)

Furthermore, the CEAC will also randomly review 25% of concordant cases for adjudication to validate the classification (Fig. 1).

### Examples of 10 scenarios observed in the trial for the adjudication process

1. SAEs with hospitalization
2. AEs with antibiotic administration
3. Blood culture—positive cases with no antibiotic treatment
4. Sepsis screen positives with no antibiotic treatment
5. Both blood culture and screen positives with no antibiotic treatment
6. Sepsis positive cases without SAE
7. Culture/sepsis positive but not PSBI by with/without sign/symptoms
8. Culture/sepsis negative with PSBI with antibiotic treatment
9. All death cases
10. Death cases after hospitalization

## Challenges in multicentric trials involving neonates

### COVID-19 pandemic

In the year 2020, the world was hit by the COVID-19 pandemic; the first cases appeared in India in March 2020, coinciding with the launch of the preparatory phase of the trial implementation. COVID-19 pandemic hampered all the trial-related activities. There was risk and fear of disease among the participant’s parents, community, and the study team. The strict government regulations of social distancing and travel restrictions were in place. This was a major limitation for site initiation, enrollment, and follow-up. In order to not deviate from the committed timelines, we decided to adopt an online mode of functioning for the preparatory phase activities such as training of staff, the site readiness/feasibility visits, the investigator’s meetings, and the site initiation visits. Under normal circumstances and as per good clinical practices, the above activities are expected to be conducted physically. This was in line with the International Council for Harmonization of Technical Requirements of Pharmaceuticals for Human Use integrated addendum to ICH E6(R1): Guideline for good clinical practice ICH E6(R3) [14]. For the conduct of the study, COVID-19 precautions/guidelines were developed and circulated to the site teams; the study staff was delegated and strictly instructed to adhere to the guidelines. The participant’s parents were informed and requested to follow the preventive measures for the disease.

However, this was challenging and required a lot of effort from participants as well as trainers to successfully conduct the implementation phase activities.

### Heterogeneity/extreme variation across sites

The trial steering committee observed variation in the morbidity pattern (adverse events, severe adverse events) across the study sites, with one outlier each in both directions. Adverse events (AEs): SMC 38.4% and JIPMER 4.4%; serious adverse events (SAEs): KEM 7.4% and AIPH 1.4%.

This could be due to the sites being systematically different from one another, e.g., due to differences in patient populations, ancillary treatment practices, or other factors [15]. Repeated quality assurance measures such as retraining of staff, review of process indicators at the sites as well as monitoring visits by the clinical trial monitors were conducted to have uniform identification and reporting of cases across all sites could not result in substantial changes in the observed morbidity pattern during trial implementation. The number of AE cases reported specifically at the JIPMER, Puducherry site and SAE cases reported at AIPH, Bhubaneswar, is lower than the other five sites, respectively.

### Regulatory and ethical committee approvals (study sites)

It was a complex and time-consuming process of obtaining multi-layered administrative, regulatory, and ethical approvals for sponsors as well as participating sites. Differences in approval requirements and timelines further cause delayed study initiation/implementation. The COVID-19 pandemic affected the EC’s functioning, resulting in sparse meetings.

The different site-specific EC requirements, variation between sites demanded several rounds of revisions and close coordination with the site investigators. The sponsor collaborated with a regulatory expert to mitigate the regulatory submission and approval challenges. The site readiness was assessed before initiation, and the CRO team catalyzed the EC submission process by actively following up with the sites.

### Recruitment challenge

There was some variability in the recruitment pattern across sites. Despite all efforts, the same pattern persisted throughout the implementation phase. Some of the primary reasons for low recruitment rate in the trial observed by us were the disruption of the obstetric services due to COVID-19 priority, lower delivery rate of LBW infants, potential participants from out of the study catchment areas, competing studies at particular sites, and lack of engagement [16]. Recruitment at SMC, Meerut site at 22.9% was the highest, and JIPMER, Puducherry 11.7% was the lowest.

Less than optimum recruitment at the study site is referred to as “research waste” in view of the time and money spent to build the site [11]. The recruitment was delayed by an average of 8.2 months from the anticipated time line, across all the study sites, and this varied from 11 to 3.5 months. A review of trials funded and published by the UK’s Health Technology Assessment program has reported that recruitment patterns in multicenter randomized trials fit more closely to Price’s Law (50% of participants are recruited by the square root of the total number of sites), than the Pareto Principle (80% of participants are recruited by 20% of sites) [16].

The trialists can hope for uniform recruitment across the sites in an ideal condition [16, 17]; however, this is difficult to achieve.

### Complex multicenter design

The randomized controlled trial (RCT) is the gold standard experimental design for assessment of interventions [4]. The multicenter trial design allows for faster recruitment over time; recruitment from different populations maximizes the generalizability [5]. However, the use of multiple clinical sites introduces complexity in clinical trials [18, 19] as they differ in geographical location, setting, distances to health care facilities, socio-economic factors, morbidity patterns, and ancillary treatment practices [20].

## Discussion

In the ICMR’s pilot trial, neonatal sepsis was detected using the Integrated Management of Neonatal and Childhood Illness (IMNCI) algorithm, a widely used clinical tool. It identifies possible serious bacterial infection (PSBI) based on a set of clinical signs. However, the study physician determined the final sepsis diagnosis after considering the clinical course, treatment received, and blood culture results (Table 1).

**Table 1:** Sepsis definitions used in ICMR’s pilot trial and the ProSPoNS trial

Definitions of sepsis used in the ICMR’s pilot trial	Definitions of sepsis in the ProSPoNS trial	Clarified definitions of sepsis in the ProSPoNS trial
• Detection of neonatal sepsis was performed during visits by field investigator, using the IMNCI algorithm for detection of the possible serious bacterial infection (PSBI). PSBI was defined as the presence of any of the following signs of infection: convulsions or fast breathing (60 breaths per minute or more); severe chest in-drawing or nasal flaring or grunting; 10 or more skin pustules or a large boil; axillary temperature 37.5 °C or above (or feels hot to touch); temperature less than 35.4 °C (or feels cold to touch); lethargic or unconscious or less than normal movements. The PSBI cases were reviewed by the site investigators. The site investigator based on his assessment, clinical course of event, treatment received, and blood culture status, decided the physician diagnosed sepsis	• Sepsis: Defined as one or more clinical signs suggestive of sepsis with a microbial isolate on blood culture or a neonate with sterile blood culture with at least two sepsis screen markers being abnormal (CRP > 12 mg/dL, absolute neutrophil count < 1500/mm^3^, TLC < 5000/mm^3^, ESR > 15 mm, immature to total neutrophil ratio > 0.2) • Possible serious bacterial infection (PSBI): One or more clinical signs—not feeding well, convulsions, severe chest in-drawing, fever (temperature ≥ 38 °C), low body temperature (< 35.5 °C), movement only when stimulated or no movement at all, fast breathing (60 breaths per minute or more) in infants less than 7 days old confirmed by the study physician	• The primary outcome of the study will be considered as physician diagnosed sepsis which includes sepsis and PSBI • The CEA committee will decide on “physician-diagnosed sepsis” based on four criteria for every event of sickness in a study participant: blood culture status, sepsis screen status, PSBI/non-PSBI signs and symptoms, and the clinical course during the event of sickness

In contrast, the ProSPoNS trial employed a more stringent definition of sepsis, which was defined as the presence of one or more clinical signs suggestive of sepsis along with positive blood culture or, in the case of sterile blood culture, at least two abnormal sepsis screen markers. However, in due course of study, it was found that these definitions were very sensitive and may have resulted in misclassification of outcomes in few cases. Also due to multicentric nature of the study, it is likely to occur across all sites and may lead to give wrong interpretation about efficacy of the intervention.

In the study protocol, hypothesis and primary objective are based on sepsis; sample size estimation has been performed based on the “physician diagnosed sepsis”; and the primary outcome has been specified as sepsis/PSBI. To align all the three above, the investigators meeting, held on 17th–18th August 2023, at MGIMS Sevagram, Wardha, deliberated and unanimously agreed the “physician diagnosed sepsis” is the primary study outcome which includes sepsis/PSBI. The CEAC’s role in the ProSPoNS trial is to determine the outcome of “physician diagnosed sepsis” based on blood culture results, sepsis screen status, clinical signs of PSBI or non-PSBI, and the clinical course during the illness. The CEA is known to reduce the misclassification and reduce the bias in outcome measurement of the study and considered as gold standard in clinical trials to achieve consistent and accurate evaluation of clinical events.

The ProSPoNS trial is a multicenter clinical trial ongoing at six sites across India. Complexity occurs in three dimensions in clinical trials: the protocol, the operations, and the potential for unanticipated change. Trials with complexity in any of these domains need special flexibility to easily adapt to the emerging variability [21]. The heterogeneity observed in the trial may be a reflection of genuine differences in the rates of neonatal sepsis between southern and northern populations of India and elsewhere as mentioned in the literature [22–25].

A review of the literature suggests considerable heterogeneity in the definitions of neonatal sepsis [26]. A related problem in these definitions is the use of subjective criteria, absence of validation leading to inability of comparison and generalizability. Most notably, there is a focus on microbiological culture for definitive diagnosis, and thereby reliance on bacteriological isolation and not sepsis per se. The definition of adult sepsis relies on multi-organ impairment and not on bacterial isolation alone [27]. A systematic review [26] identified 128 definitions from 80 RCTs after searching 688 articles. The single most common definition of neonatal sepsis was defined by blood culture alone (*n* = 35), followed by culture and clinical signs (*n* = 29), and then laboratory tests/clinical signs (*n* = 25). Blood culture featured in 83 definitions, laboratory testing featured in 48 definitions, while clinical signs and radiology featured in 80 and 8 definitions, respectively.

Additionally, we have examined the certainty of evidence from Cochrane systematic reviews on neonatal sepsis (personal communication; presented at the Cochrane colloquium 2023). Eight out of 11 Cochrane reviews reported low to very low certainty of the evidence [28] for reasons of indirectness and inconsistency, including inconsistency in how neonatal sepsis was defined in the studies [27, 29–33].

## Conclusion

In conclusion, the ProSPoNS trial, a multicenter trial being implemented across India, encountered significant challenges in its implementation and the necessity for an amendment that was unforeseen. Amid the COVID-19 pandemic, adopting online modes for training and monitoring aligned with the international guidelines posed additional challenges, necessitating considerable efforts from participants and trainers. Addressing these challenges and standardizing the definition of neonatal sepsis would ensure the validity of the trial and research outcomes. We hope that by constituting a CEAC and processing each morbidity case through their scrutiny will help mitigate misclassification and lead to unbiased measurement of the outcome.

## Data Availability

Since the study is ongoing and the adjudication is in process, the quantitative data cannot be disclosed as the data set is yet to be finalized and frozen. As soon as the data is frozen, we will publish the results. In the meantime, it will be provided on request to researchers who provide a methodically sound proposal and whose proposed use of the data has been approved by an independent review committee identified for this purpose. Such proposals may be directed to the corresponding author (apradhandr@gmail.com). However, to gain access, data requestors will need to sign and submit a cover letter mentioning the purpose with a list of requested documents along with a statement/undertaking to maintain data confidentiality.

## Abbreviations

AEs: Adverse events
AIPH: Asian Institute of Public Health
CRP: C-reactive protein
CDSCO: Central Drug Standard Control Organization
COANS: Coagulase-negative staphylococci
CRF: Case report forms
CEAC: Central Endpoint Adjudication Committee
CONMEDs: Concomitant medications
CF: Consent forms
eCRFs: Electronic case record forms
FUP: Follow-up
ICMR: Indian Council of Medical Research
JIPMER: Jawaharlal Institute of Postgraduate Medical Education and Research
KEM: King Edward’s Memorial Hospital
KSCH: Kalawati Saran Children’s Hospital
LOS: Late-onset sepsis
MGIMS: Mahatma Gandhi Institute of Medical Science
PIS: Parent information sheets
RCT: Randomized controlled trial
SAEs: Serious adverse events
SIVs: Site initiation visits
SMC: Subharti Medical College
SP: Study physician
SPF: Study physician form
TAG: The technical advisory group
TLC: Total leucocyte count
WHO: World Health Organization
UNICEF: United Nations International Children’s Emergency Fund
